# Associations of plasma concentrations of heavy metals and trace elements with estimated glomerular filtration rate and chronic kidney disease: a population-based study

**DOI:** 10.1093/ckj/sfaf383

**Published:** 2026-03-18

**Authors:** Sisi Xie, Aurélien Thomas, Belen Ponte, Daniel Ackermann, Menno Pruijm, Pedro Marques-Vidal, Murielle Bochud

**Affiliations:** Department of Medicine, Internal Medicine, Lausanne University Hospital (CHUV) and University of Lausanne, Lausanne, Switzerland; Faculty Unit of Toxicology, University Center of Legal Medicine Lausanne-Geneva, Lausanne University Hospital and University of Lausanne, Lausanne, Switzerland; Unit of Forensic Toxicology and Chemistry, CURML, Lausanne and Geneva University Hospitals, Lausanne, Geneva, Switzerland; Division of Nephrology and Hypertension, Department of Medicine, University Hospital of Geneva (HUG), Geneva, Switzerland; Department of Nephrology and Hypertension, Inselspital, Bern University Hospital and University of Bern, Bern, Switzerland; Service of Nephrology and Hypertension, Lausanne University Hospital (CHUV) and University of Lausanne, Lausanne, Switzerland; Department of Medicine, Internal Medicine, Lausanne University Hospital (CHUV) and University of Lausanne, Lausanne, Switzerland; Department of Epidemiology and Health Systems, Unisanté, Lausanne, Switzerland

**Keywords:** chronic kidney disease, eGFR, heavy, kidney function, metals, trace elements

## Abstract

**Background:**

Chronic kidney disease (CKD) is a global public health issue. Heavy metals and trace elements may influence kidney function. This study aimed to investigate the association between plasma concentrations of 24 heavy metals and trace elements and kidney function, including estimated glomerular filtration rate (eGFR) and CKD status in European adults.

**Methods:**

This study included adult participants from the Swiss Kidney Project on Genes in Hypertension cohort (2008–2013). Associations with eGFR and CKD were assessed using multivariable linear and logistic regression, and restricted cubic spline models for non-linear trends. Mixture effects were evaluated using least absolute shrinkage and selection operator for variable selection, followed by weighted quantile sum (WQS) regression. Multiple sensitivity analyses were conducted to assess robustness.

**Results:**

Of the 988 participants (mean age 47.0 years, 52.3% female), 72 (7.3%) had CKD. In multivariable linear models with continuous exposures, higher plasma tin, antimony and iodine were associated with lower eGFR, but none remained significant after false discovery rate (FDR) correction. In quartile analyses, lower eGFR was observed in the highest exposure quartile for zinc and thallium, with a borderline trend for iodine. Arsenic showed a significant non-linear relationship with eGFR, with a turning point at ≈11.7 μg/l and an estimated decline of 3.27 ml/min/1.73 m^2^ from the 25th percentile. For CKD, elevated levels of molybdenum, copper and iodine were linked to higher odds, whereas aluminum and silver were inversely associated. After FDR correction, only molybdenum remained significant. WQS regression indicated that tin, antimony, iodine, molybdenum and cadmium contributed most to lower eGFR, while iodine, arsenic, palladium, manganese and copper showed the strongest associations with CKD.

**Conclusions:**

After FDR adjustment across 24 elements, no individual element remained associated with eGFR, whereas molybdenum remained linked to CKD. Findings involving iodine were nominal and should be considered exploratory. Longitudinal and mechanistic studies are needed to validate these observations and clarify causality.

## Graphical Abstract

**Graphical Abstract:**
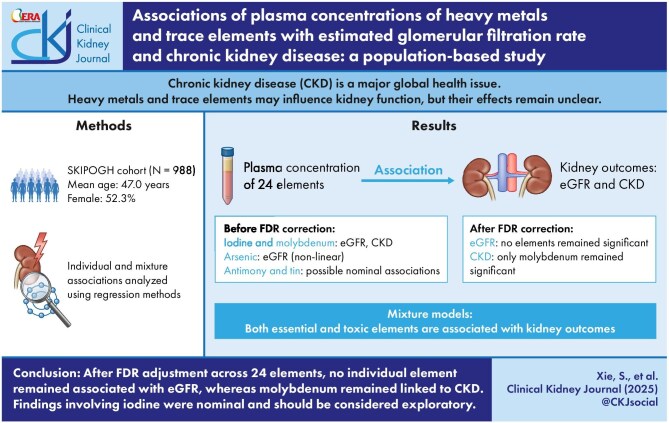

KEY LEARNING POINTS **What was known:**

- Certain heavy metals and trace elements have been linked to kidney disease, but evidence remains limited and inconsistent.
- Most studies evaluated single exposures and rarely considered non-linear or joint effects.
- Few studies used plasma biomarkers to assess internal burdens in relation to kidney function.

**This study adds:**

- After false discovery rate correction, no single-element association with eGFR persisted; the molybdenum–CKD association did persist. Signals for iodine are exploratory.
- Mixture (WQS) analyses suggest multiple elements may contribute, but these results are exploratory and supportive owing to model instability and the small number of CKD cases.
- Antimony and tin showed nominal associations with lower eGFR and warrant confirmation in longitudinal studies.

**Potential impact:**

- This study emphasizes the importance of accounting for both essential and harmful metals when studying kidney function.
- The observed associations may aid in early detection of kidney impairment.
- Findings may contribute to shaping public health strategies in kidney disease prevention.

## INTRODUCTION

Chronic kidney disease (CKD) is a disease characterized by a progressive decrease in kidney function that may ultimately lead to end-stage kidney disease requiring dialysis or kidney transplantation. CKD affects ≈10% of the adult population worldwide and increases the risk of cardiovascular disease and premature death [1]. Considering the high morbidity and mortality associated with CKD, it is crucial to identify potential risk factors for kidney dysfunction and take necessary preventive measures.

Long-term exposure to heavy metals and trace elements may result in organ damage, particularly the kidneys, which are the target organs for the excretion of these elements. Existing research shows that lead (Pb), cadmium (Cd), mercury (Hg) and arsenic (As) are common toxic heavy metals that can cause significant and irreversible damage to the kidneys [2–4]. Toxicology studies have shown that heavy metals and trace elements ultimately cause kidney function damage through a series of pathways such as oxidative stress, lipid peroxidation, mitochondrial dysfunction, damage to DNA repair mechanisms, decreased antioxidant capacity and cell apoptosis [5, 6]. Elements often coexist in the environment, and individual heavy metals and trace elements may not fully account for observed variations in kidney function. Therefore, it is worthwhile to explore their combined effects on kidney function.

In this study, we investigated the associations between 24 plasma heavy metals and trace elements and kidney function in adults from a population-based Swiss cohort. Plasma concentrations were measured using inductively coupled plasma mass spectrometry (ICP-MS) and associations were evaluated using multivariable regression approaches. We assessed both continuous estimated glomerular filtration rate (eGFR) and binary CKD outcomes to reflect complementary aspects of kidney impairment, spanning from early functional decline to established CKD. We refer to 24 heavy metals and trace elements collectively as ‘elements’ throughout the article. Given Switzerland’s rigorous environmental regulations, such as the prohibition of mercury use and strict monitoring of water quality, background exposure levels to toxic elements may be lower than in other settings, potentially influencing the observed associations.

## MATERIALS AND METHODS

### Research design and participants

The Swiss Kidney Project on Genes in Hypertension (SKIPOGH) study (www.skipogh.ch and www.maelstrom-research.org/study/skipogh) is a family-based cohort designed to assess associations between the environment, kidney function and arterial hypertension in the general population [7]. Recruited individuals were 18–90 years of age and lived in the region of the three study centres involved (Bern, Geneva and Lausanne). Biospecimens were collected at baseline from 2008 to mid-2013 and stored at −80°C until analysis. No freeze–thaw cycles occurred, minimizing pre-analytical variation.

### Measurement of 24 heavy metals and trace elements

Plasma concentrations of 24 heavy metals and trace elements were quantified using ICP-MS following a previously described protocol [8–10]. The method involves acid dilution, internal standardization using rhodium and indium, multipoint calibration and quality control with certified reference materials. Isotopes measured included ^7^Li (lithium), ^9^Be (beryllium), ^27^Al (aluminum), ^51^V (vanadium), ^53^Cr (chromium), ^55^Mn (manganese), ^59^Co (cobalt), ^60^Ni (nickel), ^63^Cu (copper), ^66^Zn (zinc), ^75^As (arsenic), ^82^Se (selenium), ^95^Mo (molybdenum), ^105^Pd (palladium), ^107^Ag (silver), ^111^Cd (cadmium), ^118^Sn (tin), ^121^Sb (antimony), ^127^I (iodine), ^195^Pt (platinum), ^201^Hg (mercury), ^205^Tl (thallium), ^208^Pb (lead) and ^209^Bi (bismuth). Supplementary Table 1 shows the full names, the number and percentage of participants with each heavy metal and trace element below the limit of detection (LOD) and the concentration distribution of heavy metals and trace elements in plasma. Element values below the LOD were calculated as the corresponding LOD divided by 2. We acknowledge that this approach may bias estimates by assigning the same value to undetected observations. Inter- and intra-assay coefficients of variation (CVs) were low for all elements, typically <5%, indicating high analytical precision. Supplementary Table 1 presents the limit of quantification (LOQ), CVs and concentrations for all 24 elements. Plasma samples were stored at −80°C from collection until analysis and did not undergo any freeze–thaw cycles, minimizing pre-analytical variation.

### Measurement of kidney function

Kidney function was assessed using eGFR and urinary albumin:creatinine ratio (UACR) in morning spot urine samples. Serum creatinine levels were measured using the Jaffe method to estimate the GFR. eGFR was calculated according to the Chronic Kidney Disease Epidemiology Collaboration (CKD-EPI) 2021 formula [11]. The distribution of baseline eGFR by Kidney Disease: Improving Global Outcomes (KDIGO) GFR stages (G-stages) is shown in Supplementary Table 2. The majority of participants had preserved kidney function (G1–G2), while only a small proportion were classified as G3a (2.5%) or G3b (0.7%). No participants were categorized as G4 or G5. CKD was defined as an eGFR <60 ml/min/1.73 m^2^ or a UACR ≥3 mg/mmol [1]. Participants with missing UACR were classified based on available eGFR data. UACR was categorized as A1 (<3 mg/mmol), A2 (3–30 mg/mmol) and A3 (>30 mg/mmol).

eGFR was estimated using the CKD-EPI 2021 creatinine equation from serum creatinine measured by the Jaffe method. The Jaffe assay may be affected by interferences (e.g. proteins, ketones, bilirubin) and tubular secretion, which could yield non-differential misclassification relative to enzymatic creatinine. Albuminuria was assessed from a single morning spot urine sample; defining CKD with a single UACR can introduce within-person variability and short-term fluctuation. To address these potential misclassification sources, we conducted prespecified sensitivity analyses.

### Covariates

We selected age, sex, education, marital status, weekly alcohol consumption, smoking, hypertension, diabetes, body mass index (BMI), C-reactive protein, 25-hydroxyvitamin D3, physical activity and study centre as covariates. BMI was categorized as a binary variable, with obesity defined as BMI ≥30 kg/m^2^ and non-obesity as BMI <30 kg/m^2^. Detailed variable definitions and measurement methods are provided in the supplementary information.

### Exclusion criteria

For this study, all SKIPOGH study participants were considered eligible. We then excluded participants with missing heavy metal and trace element data, missing eGFR data and missing at least one of the covariates defined previously.

### Ethical statement

The SKIPOGH study was approved by the ethics committees of Lausanne (CER-VD: 92/07), Geneva (CER-GE: 09-089) and Bern (CER-BE: 091/09), Switzerland. All participants provided written informed consent.

### Statistical analysis

Statistical analyses were conducted using Stata version 18 (StataCorp, College Station, TX, USA) and RStudio Desktop (version 2024.04.2+764; Posit, Boston, MA, USA). Descriptive results were expressed as the number of participants (percentage) for categorical variables and as the average ± standard deviation (SD) or median [interquartile range (IQR)] for continuous variables. Between-group comparisons were conducted using the chi-squared test for categorical variables and the Student’s *t*-test or Kruskal–Wallis test for continuous variables.

Plasma element concentrations were natural log transformed. Pearson correlations assessed pairwise associations. Multivariable regression models were used to assess associations between individual plasma element concentrations and kidney outcomes. Both eGFR (continuous) and CKD (binary) were analysed to capture complementary aspects of kidney function: eGFR reflects subclinical variations across the full population range, while CKD represents the clinically defined disease threshold.

To assess potential multicollinearity among the 24 metal and trace element exposures, we calculated variance inflation factors (VIFs) and tolerance values; all VIFs were <2 and tolerance values >0.5 (Supplementary Table 3). To enhance interpretability, all continuous regression models were estimated both per 1-unit increase in log-transformed plasma concentrations and per doubling of raw concentrations. Doubling was defined as log_2_-transformed values (i.e. ln(*x*)/ln2). For logistic regression models with CKD as the outcome, we additionally calculated the absolute risk difference (ARD) per doubling of each element, expressed in percentage points. ARD was estimated from marginal standardization of predicted probabilities using post-estimation margins in Stata. Each exposure was analysed both as a continuous variable and in quartiles. To assess linear dose–response trends across quartiles, the median concentration of each quartile was modelled as a continuous variable to derive *P* for trend values. Non-linear associations were further examined using restricted cubic spline (RCS) models with four knots placed at the 5th, 35th, 65th and 95th percentiles of the exposure distribution.

Mixture effects of multiple metals were assessed using a two-step approach. First, variable selection was performed using the least absolute shrinkage and selection operator (LASSO) regression to identify the most relevant exposures. Then, selected variables were included in a weighted quantile sum (WQS) regression model to evaluate the joint association between metal mixtures and CKD. WQS models used 1000 bootstrap iterations to ensure weight stability and both positive and negative direction models were explored. However, given the relatively small number of CKD cases, the LASSO-to-WQS pipeline may overstate signals, thus mixture results are interpreted as exploratory.

To ensure robustness, we conducted several sensitivity analyses. To mitigate potential misclassification arising from Jaffe creatinine–based eGFR and the use of a single UACR, we prespecified redefining CKD solely by eGFR (<60 ml/min/1.73 m^2^, excluding albuminuria) and modelling albuminuria as a continuous outcome (ln-transformed UACR). Additional analyses included restricting to participants with an eGFR ≥60 ml/min/1.73 m^2^, excluding the top 1% of Al and Ag, excluding elements with >10% below the LOD (to assess the impact of undetected values), applying the Benjamini–Hochberg FDR correction and using Firth logistic regression for CKD.

All models were adjusted for age, sex, education level, marital status, smoking status, alcohol consumption, physical activity, BMI (modelled as obese versus non-obese), hypertension, diabetes, vitamin D levels, C-reactive protein, physical activity and study centre. Standard errors were clustered by family code to account for familial correlations.

Statistical significance was a two-sided test with *P* < .05.

## RESULTS

### Selection of participants

We included 988 of 1129 participants, after excluding those without heavy metal and trace element concentrations (*n* = 118), eGFR (*n* = 6) and relevant covariates (*n* = 17) (Supplementary Fig. 1). Characteristics of excluded participants are shown in Supplementary Table 4. Participants in the included and excluded groups differed significantly only in terms of alcohol status and eGFR. The proportion of never-drinkers was lower in the excluded group than in the included group and their mean eGFR was also significantly lower.

Among the 988 participants included in the analysis, 52.3% were female and 72 (7.3%) had CKD. In addition, as shown in Table 1, compared with participants in the non-CKD group, participants in the CKD group were older, had hypertension more often, were diabetic, had lower education levels and had lower 25-hydroxyvitamin D_3_ levels.

**Table 1:** Baseline characteristics of study participants by CKD status, SKIPOGH study, Switzerland.

Variables	All participants (*N* = 988)	Non-CKD (*n* = 916)	CKD (*n* = 72)	*P*-value
Age (years), mean ± SD	47.0 ± 17.6	45.9 ± 17.0	60.2 ± 19.1	<.001
Female, *n* (%)	517 (52.3)	473 (51.6)	44 (61.1)	.121
Education level, *n* (%)				.002
High	360 (37.0)	346 (38.3)	14 (20.0)	
Middle	437 (44.9)	402 (44.5)	35 (50.0)	
Low	176 (18.1)	155 (17.2)	21 (30.0)	
Marital status, *n* (%)				.133
Living alone	315 (32.0)	298 (32.6)	17 (23.9)	
Living in couple	671 (68.0)	617 (67.4)	54 (76.1)	
Smoking status, *n* (%)				.495
Never	442 (45.0)	410 (45.0)	32 (45.1)	
Former	297 (30.2)	272 (29.9)	25 (35.2)	
Current	243 (24.8)	229 (25.1)	14 (19.7)	
Alcohol consumption, *n* (%)				.253
None	364 (36.9)	339 (37.0)	25 (34.7)	
1–13/week	435 (44.0)	408 (44.5)	27 (37.5)	
14–27/week	106 (10.7)	94 (10.3)	12 (16.7)	
≥28/week	83 (8.4)	75 (8.2)	8 (11.1)	
BMI group, *n* (%)				.053
Normal	856 (86.6)	799 (87.2)	57 (79.2)	
Obese	132 (13.4)	117 (12.8)	15 (20.8)	
Hypertension, *n* (%)	303 (30.8)	258 (28.2)	45 (63.4)	<.001
Diabetes, *n* (%)	42 (4.3)	33 (3.6)	9 (12.5)	<.001
Blood creatinine (μmol/l), mean ± SD	73.5 ± 14.2	72.5 ± 12.2	86.6 ± 26.3	<.001
C-reactive protein (mg/l), median (IQR)	1.5 (1.0–2.6)	1.5 (1.0–2.4)	1.3 (1.0–3.0)	.457
Vitamin D (nmol/l), mean ± SD	93.0 ± 33.8	94.1 ± 33.3	79.6 ± 37.6	.001
Urinary albumin (mg/l), median (IQR)	4.0 (1.5–8.0)	4.0 (1.5–7.0)	22.0 (8.0–59.0)	<.001
UACR categories				<.001
A1	921 (95.2)	896 (100)	25 (35.2)	
A2	42 (4.4)	0 (0)	42 (59.2)	
A3	4 (0.4)	0 (0)	4 (5.6)	
eGFR (ml/min/1.73 m^2^), mean ± SD	96.8 ± 17.9	98.4 ± 16.1	76.1 ± 25.5	<.001

Between-group comparisons were performed using the chi-squared test for categorical variables and Student’s *t*-test or Kruskal–Wallis test for continuous variables. UACR categories were based on available UACR data.

### Association between plasma element concentrations and eGFR

#### Multivariable linear regression

As shown in Table 2, in the multivariable-adjusted linear regression using continuous exposures, higher plasma concentrations of Sn, Sb and iodine were significantly associated with lower eGFR. To improve interpretability, effect estimates are shown per log-unit and per doubling of element concentration in Table 2. After FDR correction, no elements remained significant (all FDR *q* > 0.05). When element concentrations were categorized into quartiles using the lowest quartile (Q1) as the reference, significantly lower eGFR was observed among participants in the highest quartile (Q4) for Zn and Tl. Notably, the trend test for Zn was statistically significant (*P* for trend = .034), suggesting a potential dose–response relationship. Iodine also showed a borderline trend association (*P* = .052).

**Table 2:** Associations of single plasma element concentrations with eGFR. SKIPOGH study, Lausanne, Switzerland.

	Continuous				Quartiles				
Metals/trace elements	Coefficient per log unit (95% CI)	Coefficient per doubling (95% CI)	*P* value	FDR_*q*	Q1	Q2 coefficient (95% CI)	Q3 coefficient (95% CI)	Q4 coefficient (95% CI)	*P* for trend
Li	0.00 (−0.72, 0.73)	0.00 (−0.50, 0.51)	0.993	0.993	1 (ref.)	−0.02 (−2.44, 2.40)	−0.35 (−2.73, 2.02)	−1.28 (−3.85,1.29)	0.309
Be	0.44 (−0.21, 1.08)	0.30 (−0.15, 0.75)	0.187	0.360	1 (ref.)	1.35 (−0.81, 3.51)	1.80 (−0.27, 3.87)	1.28 (−0.98, 3.54)	0.224
Al	0.32 (−0.44, 1.09)	0.22 (−0.30, 0.75)	0.403	0.525	1 (ref.)	−0.85 (−2.93, 1.23)	0.34 (−1.84, 2.51)	0.92 (−1.41, 3.25)	0.296
V	2.15 (−2.15, 6.45)	1.49 (−1.49, 4.47)	0.325	0.520	1 (ref.)	0.39 (−1.95, 2.72)	1.29 (−1.10, 3.68)	0.57 (−1.97, 3.11)	0.537
Cr	0.63 (−0.29, 1.55)	0.44 (−0.20, 1.07)	0.181	0.360	1 (ref.)	−1.00 (−3.66, 1.65)	0.37 (−2.50, 3.24)	1.54 (−1.08, 4.15)	0.198
Mn	0.87 (−2.16, 3.91)	0.61 (−1.50, 2.71)	0.571	0.623	1 (ref.)	−1.06 (−3.30, 1.18)	−1.20 (−3.86, 1.46)	1.10 (−1.32, 3.52)	0.439
Co	0.54 (−0.66, 1.75)	0.38 (−0.46, 1.21)	0.373	0.525	1 (ref.)	−0.09 (−2.49, 2.31)	−1.67 (−4.20, 0.85)	−0.03 (−2.43, 2.37)	0.670
Ni	−0.54 (−1.83, 0.76)	−0.37 (−1.27, 0.53)	0.416	0.525	1 (ref.)	1.42 (−1.06, 3.90)	−1.48 (−4.31, 1.36)	−0.73 (−4.00, 2.55)	0.330
Cu	−2.59 (−6.12, 0.94)	−1.80 (−4.24, 0.65)	0.149	0.360	1 (ref.)	1.77 (−0.44, 3.99)	1.27 (−1.24, 3.78)	−0.28 (−3.38, 2.82)	0.791
Zn	−4.70 (−9.63, 0.23)	−3.26 (−6.68, 0.16)	0.061	0.346	1 (ref.)	−0.34 (−2.56, 1.89)	−1.41 (−3.64, 0.83)	**−2.47 (−4.92, −0.03)**	**0.034**
As	−0.58 (−1.29, 0.14)	−0.40 (−0.89, 0.10)	0.114	0.360	1 (ref.)	−1.85 (−4.20, 0.49)	0.02 (−2.09, 2.14)	−2.20 (−4.71, 0.31)	0.237
Se	−4.43 (−10.18, 1.33)	−3.07 (−7.06, 0.92)	0.131	0.360	1 (ref.)	−1.78 (−4.13, 0.56)	−1.23 (−3.59, 1.14)	−1.42 (−4.01, 1.18)	0.381
Mo	−1.60 (−3.71, 0.51)	−1.11 (−2.57, 0.35)	0.136	0.360	1 (ref.)	−0.07 (−2.17, 2.04)	−1.44 (−3.77, 0.90)	−1.74 (−4.11, 0.63)	0.088
Pd	−0.53 (−1.75, 0.70)	−0.36 (−1.21, 0.48)	0.397	0.525	1 (ref.)	−1.31 (−3.60, 0.97)	−2.02 (−4.62, 0.57)	−2.05 (−4.44, 0.35)	0.085
Ag	0.51 (−0.26, 1.28)	0.35 (−0.18, 0.89)	0.195	0.360	1 (ref.)	0.18 (−2.35, 2.70)	0.50 (−2.06, 3.05)	1.36 (−1.13, 3.85)	0.274
Cd	−1.53 (−3.95, 0.90)	−1.06 (−2.74, 0.62)	0.216	0.370	1 (ref.)	−0.58 (−2.82, 1.66)	−1.69 (−4.29, 0.92)	−1.07 (−3.91, 1.77)	0.351
Sn	**−0.76 (−1.46, −0.06)**	−0.53 (−1.01, −0.04)	**0.034**	0.346	1 (ref.)	−1.42 (−3.48, 0.64)	−1.60 (−4.19, 0.99)	−2.43 (−4.98, 0.13)	0.092
Sb	**−0.62 (−1.22, −0.02)**	−0.43 (−0.85, −0.01)	**0.044**	0.346	1 (ref.)	−2.02 (−4.43, 0.39)	−2.35 (−5.13, 0.43)	−1.39 (−4.26, 1.48)	0.343
I	**−1.34 (−2.66, −0.03)**	−0.93 (−1.84, −0.02)	**0.046**	0.346	1 (ref.)	−0.72 (−3.36, 1.92)	−2.51 (−5.29, 0.26)	−2.19 (−4.86, 0.48)	0.052
Pt	−0.34 (−1.49, 0.82)	−0.23 (−1.03, 0.57)	0.566	0.623	1 (ref.)	0.02 (−2.07, 2.12)	−1.47 (−3.86, 0.92)	−0.58 (−3.09, 1.93)	0.450
Hg	−0.01 (−1.38, 1.36)	−0.01 (−0.96, 0.94)	0.987	0.987	1 (ref.)	0.51 (−1.80, 2.83)	0.12 (−2.22, 2.45)	−0.11 (−2.57, 2.36)	0.858
Tl	−1.25 (−3.12, 0.62)	−0.87 (−2.16, 0.43)	0.189	0.360	1 (ref.)	−2.58 (−4.96, −0.20)	−1.10 (−3.15, 0.94)	**−2.26 (−4.50, −0.02)**	0.129
Pb	−1.13 (−2.35, 0.10)	−0.78 (−1.63, 0.07)	0.072	0.346	1 (ref.)	−1.54 (−3.82, 0.75)	−2.23 (−4.85, 0.38)	−1.87 (−4.34, 0.61)	0.119
Bi	−0.25 (−0.98, 0.49)	−0.17 (−0.68, 0.34)	0.509	0.611	1 (ref.)	0.64 (−1.93, 3.21)	−0.76 (−3.41, 1.88)	−0.35 (−2.96, 2.26)	0.552

Results are presented as regression coefficients with 95% confidence intervals, based on multivariable linear regression models. All models were adjusted for age, sex, education level, marital status, smoking status, alcohol consumption, obesity, hypertension, diabetes, vitamin D, C-reactive protein, physical activity, study center, and accounted for familial clustering using robust standard errors. The coefficient per log unit represents the association per one-unit increase in log-transformed concentration. The coefficient per doubling represents the association per doubling of concentration and was obtained by modeling log₂-transformed concentrations (log_2_[x] = ln[x]/ln[2]). FDR q-values were computed using the Benjamini–Hochberg method (N=24), and values <0.05 were considered significant. Quartile-based analyses were performed with the lowest quartile (Q1) as the reference group. P for trend was calculated by treating quartile categories as an ordinal variable in linear regression models.

#### Non-linear dose–response analysis using RCS

**Figure 1:**
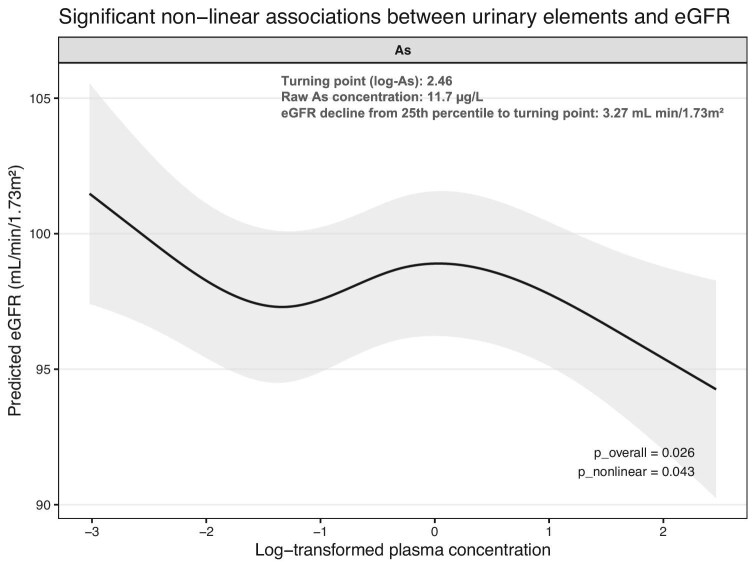
Non-linear association between plasma AS and eGFR based on RBS models. SKIPOGH study, Lausanne, Switzerland. RCS plots of log-transformed plasma concentration and predicted eGFR (ml/min/1.73 m^2^). Solid lines represent adjusted predictions and shaded areas indicate 95% CIs. *P*-values for overall and non-linear associations are shown. Turning point (log-As): 2.46; raw As concentration: 11.7 µg/l; predicted eGFR decline from 25th percentile to turning point: 3.27 ml/min/1.73 m^2^.

RCS models were applied to explore potential non-linear associations between plasma element concentrations and eGFR. As shown in Fig. 1, arsenic showed a statistically significant non-linear association with eGFR (*P* < .05 for both overall and non-linear tests), with lower eGFR observed at higher plasma concentrations, suggesting a dose-dependent decrease in kidney function. To improve interpretability, we estimated the turning point for the arsenic–eGFR relationship using the RCS model. The turning point was identified at a log-transformed arsenic concentration of 2.46, corresponding to a raw plasma concentration of ≈11.7 µg/l (≈75th percentile). From the 25th percentile to this turning point, eGFR was predicted to decrease by 3.27 ml/min/1.73 m^2^. In addition, iodine showed a borderline overall association (*P* overall = .050), although its non-linear trends were less pronounced. The remaining elements did not demonstrate statistically significant non-linear associations in spline models. Full dose–response curves for all 24 elements are presented in Supplementary Fig. 2.

#### Combined effects of plasma elements on eGFR

To assess the potential collinearity among plasma elements, we performed Pearson correlation analyses using log-transformed concentrations of all 24 heavy metals/trace elements. As shown in Supplementary Fig. 3, most pairwise correlations were weak to moderate (*r* < 0.60).

**Figure 2:**
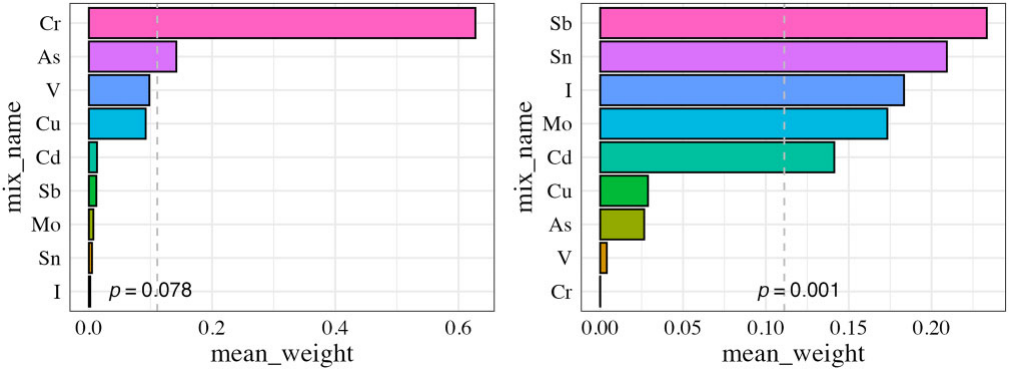
WQS regression results for the association between plasma elements and eGFR. WQS regression results show the association between nine selected heavy metals and trace elements and eGFR. The left panel represents elements with positive associations with eGFR and the right panel represents elements with negative associations. Elements were selected using LASSO regression from an initial set of 24 elements. Bars represent mean weights from WQS regression, with higher weights indicating stronger contributions.

To further evaluate the effect of combined metal exposures, we applied a two-step approach. First, LASSO regression identified nine key elements for inclusion. WQS regression was then conducted. The results showed a significant negative association between the WQS index and eGFR (*P* = .001), with Sb (mean weight 0.23), Sn (0.21), iodine (0.18), Mo (0.17) and Cd (0.14) contributing the most (weights >0.1; Fig. 2). In contrast, the positive-direction WQS model did not yield a statistically significant association with CKD (*P* = .078).

### Association between plasma element concentrations and CKD

#### Multivariable logistic regression

As shown in Table 3, in multivariable-adjusted logistic regression models using continuous exposures, higher plasma concentrations of Mo, Cu and iodine were significantly associated with increased odds of CKD. In contrast, Al and Ag were inversely associated. In addition to odds ratios (ORs) we also reported absolute risk differences per doubling of concentration, expressed as percentage point changes in CKD probability (Table 3). After FDR correction, Mo remained significant (FDR *q* < 0.05). In quartile-based analyses, higher concentrations of Mo and iodine were significantly associated with greater odds of CKD in Q4, while Ag was inversely associated in Q4. Significant associations were also observed in Q3 of iodine, Pb, Pt and Ag and Q2–Q3 of Cd, but not in Q4, suggesting potential non-linear patterns. Trend tests indicated significant monotonic associations for Mo, iodine, Ag and Al, supporting their potential relevance in CKD.

**Table 3:** Associations of single plasma element concentrations with CKD. SKIPOGH study, Lausanne, Switzerland.

	Continuous			Quartiles					
Metals/trace elements	OR (95% CI)	ARD per doubling (95% CI) % points	*P* value	FDR_*q*	Q1	Q2 OR (95% CI)	Q3 OR (95% CI)	Q4 OR (95% CI)	*P* for trend
Li	0.97 (0.78, 1.22)	−0.10 (−0.96, 0.76)	0.823	0.898	1 (ref.)	1.09 (0.47, 2.52)	1.38 (0.57, 3.32)	1.14 (0.49, 2.67)	0.689
Be	0.95 (0.77, 1.19)	−0.19 (−1.04, 0.67)	0.667	0.798	1 (ref.)	0.83 (0.33, 2.10)	0.80 (0.34, 1.84)	0.99 (0.48, 2.05)	0.962
Al	**0.77 (0.60, 0.99)**	**−1.01 (−1.98, −0.04)**	**0.041**	0.197	1 (ref.)	0.47 (0.22, 1.01)	0.56 (0.25, 1.23)	0.43 (0.18, 1.01)	**0.040**
V	0.66 (0.15, 2.83)	−1.63 (−7.28, 4.02)	0.573	0.791	1 (ref.)	1.28 (0.61, 2.69)	0.77 (0.36, 1.68)	0.90 (0.40, 2.03)	0.515
Cr	0.94 (0.72, 1.22)	−0.25 (−1.27, 0.77)	0.632	0.798	1 (ref.)	1.35 (0.61, 2.97)	1.11 (0.45, 2.72)	1.05 (0.46, 2.43)	0.852
Mn	2.77 (0.64, 11.91)	3.94 (−1.58, 9.45)	0.172	0.439	1 (ref.)	1.93 (0.73, 5.13)	1.46 (0.52, 4.08)	2.07 (0.69, 6.22)	0.268
Co	1.14 (0.81, 1.59)	0.50 (−0.81, 1.81)	0.453	0.777	1 (ref.)	0.98 (0.45, 2.12)	1.18 (0.49, 2.86)	1.57 (0.74, 3.31)	0.172
Ni	1.22 (0.85, 1.76)	0.78 (−0.61, 2.17)	0.274	0.548	1 (ref.)	0.43 (0.16, 1.17)	1.26 (0.57, 2.78)	1.28 (0.49, 3.38)	0.497
Cu	**4.70 (1.20, 18.37)**	**5.96 (0.63, 11.29)**	**0.026**	0.186	1 (ref.)	0.82 (0.32, 2.08)	1.04 (0.42, 2.58)	1.74 (0.65, 4.64)	0.188
Zn	0.97 (0.11, 8.65)	−0.13 (−8.63, 8.38)	0.976	0.976	1 (ref.)	0.76 (0.34, 1.71)	0.79 (0.36, 1.72)	0.83 (0.32, 2.16)	0.676
As	1.22 (0.99, 1.49)	0.75 (−0.03, 1.53)	0.058	0.232	1 (ref.)	0.87 (0.33, 2.26)	1.19 (0.47, 3.02)	1.76 (0.76, 4.06)	0.107
Se	0.31 (0.05, 1.74)	−4.57 (−11.16, 2.02)	0.183	0.439	1 (ref.)	1.10 (0.57, 2.12)	0.66 (0.27, 1.58)	0.64 (0.26, 1.59)	0.222
Mo	**2.59 (1.40, 4.79)**	**3.63 (1.23, 6.04)**	**0.002**	**0.048**	1 (ref.)	0.93 (0.39, 2.22)	0.81 (0.35, 1.90)	**2.10 (1.04, 4.23)**	**0.048**
Pd	1.36 (0.89, 2.08)	1.18 (−0.49, 2.85)	0.162	0.439	1 (ref.)	1.20 (0.40, 3.57)	1.67 (0.60, 4.65)	1.75 (0.69, 4.43)	0.187
Ag	**0.75 (0.58, 0.97)**	**−1.11 (−2.14, −0.09)**	**0.031**	0.186	1 (ref.)	0.53 (0.23, 1.24)	**0.44 (0.20, 0.97)**	**0.41 (0.18, 0.92)**	**0.040**
Cd	1.25 (0.63, 2.47)	0.86 (−1.78, 3.50)	0.524	0.791	1 (ref.)	**3.23 (1.18, 8.85)**	**3.84 (1.22, 12.04)**	2.33 (0.75, 7.25)	0.405
Sn	0.92 (0.70, 1.21)	−0.33 (−1.40, 0.74)	0.548	0.791	1 (ref.)	0.73 (0.29, 1.86)	0.70 (0.28, 1.72)	0.66 (0.26, 1.66)	0.395
Sb	1.27 (0.93, 1.74)	0.93 (−0.24, 2.09)	0.133	0.439	1 (ref.)	0.96 (0.33, 2.82)	0.92 (0.32, 2.65)	1.43 (0.48, 4.25)	0.519
I	**2.01 (1.19, 3.39)**	**2.64 (0.60, 4.67)**	**0.009**	0.108	1 (ref.)	1.78 (0.78, 4.09)	**2.66 (1.16, 6.09)**	**3.56 (1.53, 8.30)**	**0.002**
Pt	1.20 (0.85, 1.69)	0.71 (−0.63, 2.05)	0.297	0.548	1 (ref.)	1.41 (0.60, 3.35)	**2.33 (1.06, 5.13)**	1.25 (0.50, 3.08)	0.279
Hg	0.75 (0.48, 1.18)	−1.12 (−2.85, 0.61)	0.210	0.458	1 (ref.)	1.50 (0.66, 3.43)	0.77 (0.33, 1.78)	0.73 (0.30, 1.79)	0.213
Tl	0.89 (0.51, 1.58)	−0.44 (−2.65, 1.77)	0.698	0.798	1 (ref.)	1.56 (0.80, 3.06)	0.83 (0.37, 1.86)	1.02 (0.47, 2.23)	0.784
Pb	1.03 (0.75, 1.41)	0.10 (−1.13, 1.34)	0.868	0.906	1 (ref.)	1.17 (0.48, 2.90)	**2.51 (1.08, 5.81)**	1.27 (0.55, 2.94)	0.424
Bi	1.06 (0.86, 1.31)	0.22 (−0.59, 1.03)	0.593	0.791	1 (ref.)	0.76 (0.30, 1.89)	1.05 (0.47, 2.33)	1.36 (0.66, 2.78)	0.362

Results are expressed as odds ratios (OR) with 95% confidence intervals (CI). Logistic regression models were adjusted for age, sex, education level, marital status, smoking, alcohol consumption, physical activity, obesity, hypertension, diabetes, vitamin D, C-reactive protein, physical activity, study center, and accounted for familial clustering using robust standard errors. The continuous model represents the association per one-unit increase in log-transformed concentration. Absolute Risk Difference (ARD) per doubling was obtained from models using log₂-transformed concentrations (log₂[x] = ln[x]/ln[2]) and estimated using marginal effects (margins in Stata), expressed as absolute risk difference (percentage points). FDR q-values were calculated using the Benjamini–Hochberg procedure to correct for multiple comparisons (N = 24); q < 0.05 was considered statistically significant. Quartile models compare each quartile (Q2–Q4) to the reference (Q1), and p for trend was calculated by modeling the median value of each quartile as a continuous variable.

#### Non-linear dose–response analysis using RCS

RCS models were used to evaluate potential non-linear associations between plasma element concentrations and CKD. No statistically significant non-linear relationships were observed for any of the elements (all *P* > .05). For completeness, dose–response curves for all 24 elements are presented in Supplementary Fig. 4.

#### Combined effects of plasma heavy metals and trace elements on CKD

As previously described, Pearson correlation analyses showed that most pairwise correlations among plasma elements were weak to moderate (*r* < 0.60), suggesting acceptable collinearity for combined exposure modelling.

**Figure 3:**
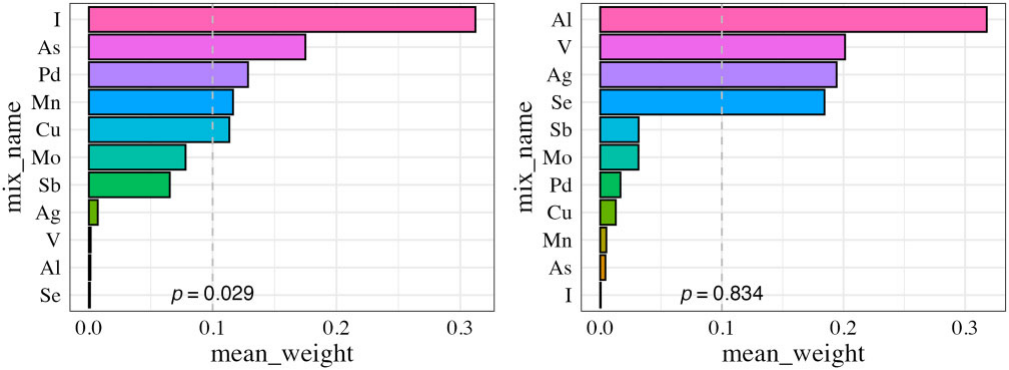
WQS regression results for the association between plasma elements and CKD. WQS regression results show the association between 11 selected heavy metals and trace elements and CKD. The left panel represents elements with positive associations with CKD and the right panel represents elements with negative associations. Elements were selected using LASSO regression from an initial set of 24 elements. Bars represent mean weights from WQS regression, with higher weights indicating stronger contributions.

We also applied a two-step approach. First, LASSO regression identified 11 key elements for inclusion. WQS regression was then conducted. The results showed a significant positive association between WQS index and CKD events (*P* = .029), among which iodine (mean weight 0.31), As (0.17), Pd (0.13), Mn (0.12) and Cu (0.11) showed weighted values >0.1 (Fig. 3). In contrast, the negative-direction WQS model did not yield a statistically significant association with CKD (*P* = .834).

### Sensitivity analyses

#### Sensitivity analyses for eGFR models

To evaluate whether associations were similar among participants with preserved kidney function, we restricted the analysis to those with an eGFR ≥60 ml/min/1.73 m^2^ (*n* = 956). As shown in Supplementary Table 5, the inverse associations of Zn and Sn with eGFR remained statistically significant, in line with the main analysis. For albuminuria (log-UACR), positive associations were observed for Mo and iodine, further supporting their potential relevance in early kidney dysfunction even in the absence of overt CKD.

#### Sensitivity analyses for CKD models

Given the limited number of CKD cases (*n* = 72), we conducted additional sensitivity analyses to test the robustness of the observed associations. First, we applied Firth logistic regression to reduce small-sample bias; results for elements such as Al, Cu, Mo and iodine were directionally similar to the main analysis (Supplementary Table 6). Second, we redefined CKD based solely on eGFR <60 ml/min/1.73 m^2^ (excluding albuminuria), and the associations for Mo and iodine remained significant (Supplementary Table 7). These findings support the robustness of the primary findings.

#### Sensitivity analyses for Al and Ag

Given concerns regarding the inverse associations observed for Al and Ag, we conducted additional analyses to evaluate their stability. First, we excluded the top 1% of plasma Al and Ag values to reduce the influence of potential outliers. As shown in Supplementary Table 8, the association between Al and CKD became non-significant {OR 0.78 [95% confidence interval (CI) 0.60–1.01], *P* = .061}, while the association for Ag remained significant [OR 0.69 (95% CI 0.54–0.88), *P* = .003]. Second, we excluded all elements with >10% of values below the LOD, which includes Al, Ag, Sn, Pt and Bi. As shown in Supplementary Table 9, the associations for both Al and Ag were attenuated and no longer statistically significant. These results suggest that the initial inverse associations may have been influenced by outlier values or measurement limitations and should be interpreted with caution.

#### Sensitivity analysis excluding elements with low detection rates

To assess the potential influence of low detection rates on the robustness of associations, we conducted a sensitivity analysis excluding plasma elements with >10% of values below the LOD. As shown in Supplementary Table 9, the associations for well-detected elements were generally similar in direction and magnitude. In contrast, the initially observed inverse associations between Al and CKD, as well as Ag and CKD, were attenuated and lost statistical significance, indicating potential instability. These findings suggest that results involving poorly detected elements should be interpreted with caution.

## DISCUSSION

In this population-based cross-sectional study, several plasma elements showed nominal associations with kidney function. Higher Sn, Sb and iodine were linked with lower eGFR; Zn and Tl were inversely associated only at higher exposure quartiles; and arsenic displayed a non-linear inverse pattern. However, after controlling the FDR across 24 elements, no single-element association with eGFR persisted, whereas Mo remained associated with CKD. Signals involving iodine should therefore be considered exploratory. In mixture analyses, WQS suggested contributions from multiple elements (notably Sb, Sn, iodine, Mo, Cd and As), but we interpret these findings as exploratory and supportive given model instability and the limited number of CKD cases; nonetheless, the directions were broadly consistent with single-element patterns.

### Toxic elements and potential mechanisms

#### Cd

Prior studies support Cd nephrotoxicity and higher CKD risk [12–14]. In our study, plasma Cd concentrations were associated with increased odds of CKD, particularly in intermediate exposure quartiles, and contributed notably to lower eGFR in mixture models, consistent with mechanisms involving oxidative stress, impaired DNA repair and apoptosis [15–17].

#### As

As has also been implicated in kidney dysfunction in multiple studies [18–20]. In our analysis, As contributed substantially to the WQS indices for both eGFR and CKD and showed a significant non-linear inverse association with eGFR. The turning point in the spline models (11.7 μg/l) falls within ranges observed in general populations and suggests a biologically relevant threshold. This turning point should be viewed as an illustrative feature of the exposure–response curve rather than a clinical threshold. Experimental and clinical evidence links As exposure to tubular injury and oxidative stress [21].

#### Sb and Sn

Sb and Sn, which contributed notably in WQS models of eGFR, are less well characterized in nephrotoxicity research. Sb (III) experiments show loss of epithelial viability, profibrotic gene induction and ferroptosis-related oxidative/iron dysregulation [22, 23]. Moreover, epidemiologic data have linked urinary Sb concentrations with increased UACR, but links to eGFR remain uncertain [24]. For Sn, animal data on organotin indicate renal accumulation, oxidative stress, inflammation, apoptosis and reduced filtration [25, 26]. The association persisted in analyses restricted to participants with preserved eGFR, indicating a possible role of Sn in early kidney impairment.

#### Other metals (Pb, Pt, Tl, Pd)

We observed isolated quartile-specific signals, compatible with threshold or non-linear effects. Given the limited prior evidence, especially for Pd, these findings should be interpreted cautiously.

### Essential elements and potential mechanisms

#### Iodine

Iodine is an essential trace element required for the synthesis of thyroid hormones. In our study, higher plasma iodine was associated with lower eGFR and higher CKD odds and was a key WQS contributor. Although the underlying mechanisms remain incompletely understood, animal studies have demonstrated that chronic iodine excess can cause kidney structural abnormalities and functional impairment [27]. One hypothesis is that elevated iodine levels may reflect higher dietary salt intake, as iodized salt is the main iodine source in Switzerland, accounting for ≈44% of total iodine intake [28]. However, in our study, plasma iodine levels were not significantly correlated with 24-hour urinary sodium excretion (data not shown), suggesting that the observed associations are unlikely to be confounded by salt intake alone. These associations were directionally consistent across sensitivity analyses, supporting robustness, but should be interpreted as exploratory.

#### Mo

Mo is an essential cofactor for several enzymes involved in redox reactions and purine metabolism [29]. In our study, higher plasma Mo was associated with greater CKD odds across continuous and quartile models, with a monotonic pattern supporting a possible dose response. Excessive Mo intake has been associated with altered purine metabolism and increased oxidative stress, promoting kidney injury. Prior work aligns with this signal: Liu *et al*. [31] reported a significant association between elevated plasma Mo levels and kidney function decline in a prospective cohort study, and a paediatric CKD study [32] reported elevated Mo, with 46% above reference, and an inverse correlation with eGFR. Findings for Mo were generally consistent across sensitivity analyses, reinforcing the robustness of this association.

#### Cu and Zn

Cu is an essential trace element involved in various enzymatic and redox reactions [33]. In our analysis, Cu was significantly associated with increased CKD prevalence and contributed notably in the WQS mixture model. While physiologic Cu is necessary, excess may induce oxidative stress and inflammation, pathways implicated in renal injury [34]. Defining the threshold between adequate and harmful levels warrants study. Zn showed an inverse association with eGFR in the highest quartile, with a positive trend across quartiles; experimental work suggests high Zn intake reduces kidney blood flow via oxidative suppression of nitric oxide [35], compatible with U-shaped or threshold effects. This pattern remained consistent in participants with preserved eGFR.

#### Al and Ag

In addition, both Al and Ag exhibited inverse associations with CKD. Given the limited and conflicting evidence regarding their roles in kidney physiology, these findings should be interpreted with caution and considered exploratory. Sensitivity analyses further indicated instability in these associations. After excluding extreme values and elements with a high proportion of results below the LOD, the inverse associations attenuated and became non-significant, suggesting possible measurement imprecision or the influence of outliers.

### Strengths and limitations

This study has several notable strengths. First, it was conducted in a population-based Swiss cohort with standardized recruitment and biobanking procedures, enhancing internal validity and sample integrity. Second, plasma concentrations of 24 heavy metals and trace elements were measured using high-precision ICP-MS, with low inter- and intra-assay variability. Third, we performed extensive sensitivity analyses, redefining CKD, excluding outliers and poorly detected elements and applying multiple statistical corrections, to ensure the robustness of our findings (Supplementary Tables 5–9).

However, several limitations should be acknowledged. First, the cross-sectional design limits causal inference and reverse causation cannot be excluded. The current creatinine-based results should therefore be regarded as exploratory. Second, residual confounding is possible, particularly given the lack of detailed dietary and thyroid function data. In particular, the absence of comprehensive dietary information for iodine, As and Mo may lead to potential exposure misclassification, as dietary sources and bioavailability of these elements were not fully characterized. This represents a dominant limitation when interpreting associations driven by nutritional versus environmental pathways. Third, eGFR was derived from Jaffe creatinine and CKD was partly defined using a single UACR, which may introduce non-differential misclassification and bias estimates toward the null. Still, it would be difficult to conduct several creatinine assessments in an epidemiological setting. We therefore highlighted sensitivity analyses—eGFR-only CKD and continuous ln(UACR)—that were consistent with the main results. Fourth, the number of CKD cases was relatively small, although associations with eGFR were robust and Firth regression yielded similar inferences. Fifth, several elements (e.g. Al, Ag) had substantial proportions of values below the LOD. We used LOD/2 substitution, which may introduce bias, therefore results involving poorly detected elements were interpreted with caution. Familial clustering was accounted for by clustering standard errors by family code (275 families, 988 participants). Although this approach provides robust inference, it does not explicitly capture shared familial traits and is acknowledged as a limitation. Finally, findings from a low-exposure Swiss context may not fully generalize to populations with higher exposures. Cystatin C–based analyses will be pursued in future work.

### Public health and translational implications

Although exploratory, our findings suggest that systematic surveillance of environmental and dietary exposures could help identify modifiable contributors to kidney disease and inform prevention. These signals are not yet clinically actionable, but they warrant validation in longitudinal cohorts and evaluation of exposure-reduction strategies in high-risk groups.

### Differences between modelling approaches

Some elements (e.g. Sn, Sb, iodine) were associated with lower eGFR in continuous models but not in quartile-based analyses, while Zn and Tl showed inverse associations only in the highest quartile. These discrepancies may reflect non-linear or threshold effects, differences in power or sensitivity to cut-offs. Continuous models assume linearity, whereas quartile models are more flexible but less precise. Several elements related to eGFR did not translate to CKD, consistent with the outcome scale: eGFR (continuous) captures subtle change, whereas CKD is dichotomous with few cases, reducing power and reflecting different biology from albuminuria. Sensitivity analyses using alternative CKD definitions and Firth regression yielded consistent directions, supporting robustness.

## CONCLUSION

In this population-based cross-sectional study, several plasma elements showed nominal associations with kidney outcomes. After FDR correction, no single-element association with eGFR persisted, whereas Mo remained associated with CKD. Findings involving iodine are exploratory. Given the cross-sectional design, causal inference is not possible; longitudinal and mechanistic studies are needed to validate these observations.

## Supplementary Material

sfaf383_Supplemental_File

## Data Availability

SKIPOGH data can be made available upon request to the SKIPOGH investigators as specified in the Maelström Catalogue (Maelstrom Research, Montreal, QC, Canada).

## References

[1] Kidney Disease: Improving Global Outcomes CKD Work Group . KDIGO 2024 clinical practice guideline for the evaluation and management of chronic kidney disease. Kidney Int 2024;105(4 Suppl):S117–314. 10.1016/j.kint.2023.10.018 38490803

[2] Sanders AP, Mazzella MJ, Malin AJ et al. Combined exposure to lead, cadmium, mercury, and arsenic and kidney health in adolescents age 12–19 in NHANES 2009–2014. Environ Int 2019;131:104993. 10.1016/j.envint.2019.104993 31326826 PMC6750805

[3] Grünfeld JP, Rossier BC. Lithium nephrotoxicity revisited. Nat Rev Nephrol 2009;5:270–6. 10.1038/nrneph.2009.43 19384328

[4] Yin G, Xin M, Zhao S et al. Heavy metals and elderly kidney health: a multidimensional study through Enviro-target Mendelian Randomization. Ecotoxicol Environ Saf 2024;281:116659. 10.1016/j.ecoenv.2024.116659 38964060

[5] Tchounwou PB, Yedjou CG, Patlolla AK et al. Heavy metal toxicity and the environment. Exp Suppl 2012;101:133–64. 22945569 10.1007/978-3-7643-8340-4_6 PMC4144270

[6] Mishra M, Nichols L, Dave AA et al. Molecular mechanisms of cellular injury and role of toxic heavy metals in chronic kidney disease. Int J Mol Sci 2022;23:11105. 10.3390/ijms231911105 36232403 PMC9569673

[7] Stalder E, Haldimann M, Blanc A et al. Use of day and night urinary iodine excretion to estimate the prevalence of inadequate iodine intakes via the estimated average requirement cut-point method. Swiss Med Wkly 2019;149:w20090. 10.4414/smw.2019.20090 31154659

[8] Perrais M, Trächsel B, Lenglet S et al. Reference values for plasma and urine trace elements in a Swiss population-based cohort. Clin Chem Lab Med 2024;62:2242–55. 10.1515/cclm-2023-1433 38641868

[9] Jafari P, Thomas A, Haselbach D et al. Trace element intakes should be revisited in burn nutrition protocols: a cohort study. Clin Nutr 2018;37:958–64. 10.1016/j.clnu.2017.03.028 28455105

[10] Xie S, Perrais M, Golshayan D et al. Association between urinary heavy metal/trace element concentrations and kidney function: a prospective study. Clin Kidney J 2025;18:sfae378. 10.1093/ckj/sfae378 39950154 PMC11822291

[11] Inker LA, Eneanya ND, Coresh J et al. New creatinine- and cystatin C-based equations to estimate GFR without race. N Engl J Med 2021;385:1737–49. 10.1056/NEJMoa2102953 34554658 PMC8822996

[12] Akinleye A, Oremade O, Xu X. Exposure to low levels of heavy metals and chronic kidney disease in the US population: a cross sectional study. PLoS One 2024;19:e0288190. 10.1371/journal.pone.0288190 38625896 PMC11020388

[13] Madrigal JM, Ricardo AC, Persky V et al. Associations between blood cadmium concentration and kidney function in the U.S. population: impact of sex, diabetes and hypertension. Environ Res 2019;169:180–8. 10.1016/j.envres.2018.11.009 30466011 PMC6347526

[14] Wang D, Sun H, Wu Y et al. Tubular and glomerular kidney effects in the Chinese general population with low environmental cadmium exposure. Chemosphere 2016;147:3–8. 10.1016/j.chemosphere.2015.11.069 26751126

[15] Nair AR, Lee WK, Smeets K et al. Glutathione and mitochondria determine acute defense responses and adaptive processes in cadmium-induced oxidative stress and toxicity of the kidney. Arch Toxicol 2015;89:2273–89. 10.1007/s00204-014-1401-9 25388156

[16] Bork U, Lee WK, Kuchler A et al. Cadmium-induced DNA damage triggers G_2_/M arrest via chk1/2 and cdc2 in p53-deficient kidney proximal tubule cells. Am J Physiol Renal Physiol 2010;298:F255–65. 10.1152/ajprenal.00273.2009 19923412

[17] Kim HR, Lee KY, Ahn SG et al. Transcriptional regulation, stabilization, and subcellular redistribution of multidrug resistance-associated protein 1 (MRP1) by glycogen synthase kinase 3αβ: novel insights on modes of cadmium-induced cell death stimulated by MRP1. Arch Toxicol 2015;89:1271–84. 10.1007/s00204-014-1381-9 25273023

[18] Zuo J, Huesker K, Liu Y et al. Association of whole blood heavy metal concentrations with kidney function. Sci Rep 2025;15:8370. 10.1038/s41598-025-93548-7 40069484 PMC11897145

[19] McKeon HP, Chen W, Te Biesebeek JD et al. Urinary concentrations of arsenic species in older Dutch adults and risk of chronic kidney disease. Environ Int 2025;196:109289. 10.1016/j.envint.2025.109289 39923487

[20] Chen HH, Huang YL, Wu CY et al. Plasma myeloperoxidase interactions with cadmium, lead, arsenic, and selenium and their impact on chronic kidney disease. Ecotoxicol Environ Saf 2025;290:117726. 10.1016/j.ecoenv.2025.117726 39826409

[21] Orr SE, Bridges CC. Chronic kidney disease and exposure to nephrotoxic metals. Int J Mol Sci 2017;18:1039. 10.3390/ijms18051039 28498320 PMC5454951

[22] Roldán N, Verdugo M, Suzuki N et al. Toxicological effects of Sb(III), Sb(V), and NMG-Sb(V) in human lung, kidney, and liver cells: cytotoxicity and fibrotic factor induction. J Toxicol Sci 2025;50:283–92. 10.2131/jts.50.283 40451857

[23] Liu L, Luo C, Zheng D et al. TRPML1 contributes to antimony-induced nephrotoxicity by initiating ferroptosis via chaperone-mediated autophagy. Food Chem Toxicol 2024;184:114378. 10.1016/j.fct.2023.114378 38097005

[24] Grau-Perez M, Domingo-Relloso A, Garcia-Barrera T et al. Association of single and joint metals with albuminuria and estimated glomerular filtration longitudinal change in middle-aged adults from Spain: the Aragon workers health study. Environ Pollut 2023;318:120851. 10.1016/j.envpol.2022.120851 36509352

[25] Barbosa CML, Ferrão FM, Graceli JB. Organotin compounds toxicity: focus on kidney. Front Endocrinol 2018;9:256. 10.3389/fendo.2018.00256 PMC5972511 29872423

[26] Coutinho JV, Freitas-Lima LC, Freitas FF et al. Tributyltin chloride induces renal dysfunction by inflammation and oxidative stress in female rats. Toxicol Lett 2016;260:52–69. 10.1016/j.toxlet.2016.08.007 27521499

[27] Guo Y, Hu C, Xia B et al. Iodine excess induces hepatic, renal and pancreatic injury in female mice as determined by attenuated total reflection Fourier-transform infrared spectrometry. J Appl Toxicol 2022;42:600–16. 10.1002/jat.4242 34585417

[28] Haldimann M, Bochud M, Burnier M et al. Prevalence of iodine inadequacy in Switzerland assessed by the estimated average requirement cut-point method in relation to the impact of iodized salt. Public Health Nutr 2015;18:1333–42. 10.1017/S1368980014002018 25231207 PMC10271515

[29] Hille R, Nishino T, Bittner F. Molybdenum enzymes in higher organisms. Coord Chem Rev 2011;255:1179–205. 10.1016/j.ccr.2010.11.034 21516203 PMC3079273

[30] Chung HY, Baek BS, Song SH et al. Xanthine dehydrogenase/xanthine oxidase and oxidative stress. AGE 1997;20:127–40. 10.1007/s11357-997-0012-2 23604305 PMC3455892

[31] Liu Y, Yuan Y, Xiao Y et al. Associations of plasma metal concentrations with the decline in kidney function: a longitudinal study of Chinese adults. Ecotoxicol Environ Saf 2020;189:110006. 10.1016/j.ecoenv.2019.110006 31812020

[32] Filler G, Belostotsky V, Kobrzynski M et al. High prevalence of elevated molybdenum levels in pediatric CKD patients. A cross-sectional and longitudinal study. Clin Nephrol 2017;88:79–85. 28502322 10.5414/CN109015

[33] Kim BE, Nevitt T, Thiele DJ. Mechanisms for copper acquisition, distribution and regulation. Nat Chem Biol 2008;4:176–85. 10.1038/nchembio.72 18277979

[34] Vo TTT, Peng TY, Nguyen TH et al. The crosstalk between copper-induced oxidative stress and cuproptosis: a novel potential anticancer paradigm. Cell Commun Signal 2024;22:353. 10.1186/s12964-024-01726-3 38970072 PMC11225285

[35] Yanagisawa H, Miyazaki T, Nodera M et al. Zinc-excess intake causes the deterioration of renal function accompanied by an elevation in systemic blood pressure primarily through superoxide radical-induced oxidative stress. Int J Toxicol 2014;33:288–96. 10.1177/1091581814532958 24808049

