# Supplementary material for: Associations of plasma concentrations of heavy metals and trace elements with estimated glomerular filtration rate and chronic kidney disease: a population-based study
Source: Clin Kidney J. 2026 Mar 18;19(4):sfaf383. doi: 10.1093/ckj/sfaf383 (PMC13044714; doi:10.1093/ckj/sfaf383)
Supplement: sfaf383_Supplemental_File [file sfaf383_supplemental_file.docx]

**Supplementary Materials**

**Supplementary methods:**

**Covariate definitions**

We selected age (years), sex (male/female), education (low/medium/high), marital status (living alone/living in couple), weekly alcohol consumption (units), smoking (never/former/current), hypertension (yes/no), diabetes (yes/no), body mass index (BMI) (normal/obese), C-reactive protein (continuous), 25-hydroxyvitamin D3, physical activity (low/moderate/high), and study center (Lausanne, Geneva, and Bern).

Education and marital status were categorized based on questionnaire responses. Usual alcohol consumption during the week was self-reported and reported as the number of units (glasses of wine, bottles or cans of beer, and shots of spirits) per week. Smoking was self-reported and categorized as never, former (irrespective of the time since quitting smoking), and current. Physical activities were categorized as low, moderate, and high, based on weekly duration.

BMI was categorized as a binary variable, with obesity defined as BMI ≥30 kg/m², and non-obesity as BMI <30 kg/m². Hypertension was defined by elevated office blood pressure (SBP ≥140 mm Hg or DBP ≥90 mm Hg) or use of antihypertensive medication. Diabetes was defined as fasting glucose ≥7.0 mmol/L or the use of antidiabetic medication. Plasma 25-hydroxyvitamin D3 was measured by a direct, competitive [chemiluminescence immunoassay](https://www.sciencedirect.com/topics/medicine-and-dentistry/chemiluminescence-immunoassay) on a LIAISON analyzer (DiaSorin S.p.A., Saluggia, Italy). The intra- and inter-assay coefficients of variation are 6.6% to 7.1% for plasma 25-hydroxyvitamin D3. CRP was measured using high high-sensitivity immunoturbidimetric assay.

**BMI sensitivity analysis:**

Modeling BMI as a continuous variable rather than binary (obese vs. non-obese) for all 24 elements yielded estimates similar in direction and magnitude for both eGFR and CKD outcomes, with the same elements remaining significant. No conclusions changed (data not shown).

**Supplementary Table 1:** Abbreviations, full names, percentile distribution of 24 heavy metals/trace elements, and concentration, number, and percentage of below limit of detection LOD. SKIPOGH study, Lausanne, Switzerland.

| **Abbreviations** | **Full names** | **Median** | **Interquartile Range** | **LOD**  **(µg/L)** | **Number of observations below LOD (n and %)** | | **LOQ**  **(µg/L)** | **CVr**  **(%)** | **CVR**  **(%)** | **Conc (µg/L)** |
| --- | --- | --- | --- | --- | --- | --- | --- | --- | --- | --- |
| Li | Lithium | 1.40 | 0.69-2.81 | 0.437 | 157 | 0.621 | 0.621 | 4.20% | 5.50% | 318 |
| Be | Beryllium | 0.002 | 0.001-0.005 | 0.001 | 351 | 0.004 | 0.004 | 3.50% | 6.10% | 0.954 |
| Al | Aluminum | 1.39 | 1.00-2.50 | 1.943 | 635 | 4.489 | 4.489 | 3.20% | 3.40% | 55.6 |
| V | Vanadium | 0.24 | 0.22-0.28 | 0.010 | 0 | 0.026 | 0.026 | 2.90% | 4.30% | 1.11 |
| Cr | Chromium | 0.34 | 0.13-0.72 | 0.189 | 391 | 0.559 | 0.559 | 3.30% | 4.50% | 3.19 |
| Mn | Manganese | 0.53 | 0.45-0.63 | 0.123 | 0 | 0.377 | 0.377 | 3.90% | 7.90% | 4.45 |
| Co | Cobalt | 0.10 | 0.08-0.15 | 0.003 | 0 | 0.015 | 0.015 | 1.80% | 4.50% | 2.21 |
| Ni | Nickel | 1.09 | 0.82-1.78 | 0.433 | 118 | 1.070 | 1.070 | 1.70% | 4.70% | 5.72 |
| Cu | Copper | 952.44 | 813.51-1123.76 | 1.147 | 0 | 3.479 | 3.479 | 1.70% | 3.10% | 692 |
| Zn | Zinc | 784.12 | 708.59-863.39 | 0.421 | 0 | 5.536 | 5.536 | 1.20% | 3.90% | 1160 |
| As | Arsenic | 0.46 | 0.20-1.11 | 0.069 | 36 | 0. 167 | 0. 167 | 1.50% | 2.50% | 9.82 |
| Se | Selenium | 114.82 | 102.31-128.39 | 0.614 | 0 | 1.680 | 1.680 | 1.40% | 2.50% | 81.4 |
| Mo | Molybdenum | 0.88 | 0.70-1.09 | 0.023 | 0 | 0.074 | 0.074 | 2.00% | 2.80% | 1.77 |
| Pd | Palladium | 0.08 | 0.06-0.11 | 0.009 | 1 | 0.018 | 0.018 | 2.30% | 3.40% | 2.04 |
| Ag | Silver | 0.19 | 0.09-0.41 | 0.100 | 258 | 0.100 | 0.100 | 2.60% | 2.80% | 1.00 |
| Cd | Cadmium | 0.05 | 0.04-0.07 | 0.013 | 2 | 0.045 | 0.045 | 8.20% | 9.40% | 0.232 |
| Sn | Tin | 0.16 | 0.06-0.27 | 0.036 | 140 | 0.129 | 0.129 | 3.50% | 3.80% | 1.29 |
| Sb | Antimony | 0.03 | 0.02-0.04 | 0.003 | 59 | 0.015 | 0.015 | 1.40% | 1.70% | 2.53 |
| I | Iodine | 57.49 | 40.30-70.69 | 0.725 | 3 | 1.808 | 1.808 | 1.40% | 4.60% | 45.8 |
| Pt | Platinum | 0.03 | 0.02-0.03 | 0.020 | 254 | 0.040 | 0.040 | 8.80% | 8.80% | 0.05 |
| Hg | Mercury | 0.27 | 0.18-0.41 | 0.069 | 17 | 0.213 | 0.213 | 2.80% | 9.50% | 2.05 |
| Tl | Thallium | 0.04 | 0.03-0.04 | 0.003 | 0 | 0.007 | 0.007 | 1.50% | 2.60% | 0.525 |
| Pb | Lead | 1.08 | 0.69-1.61 | 0.070 | 1 | 0.245 | 0.245 | 6.50% | 9.90% | 0.344 |
| Bi | Bismuth | 0.01 | 0.005-0.015 | 0.004 | 231 | 0.010 | 0.010 | 2.10% | 4.30% | 0.092 |

LOD: Limit of Detection (Ratio + 3 SD)/a; LOQ: Limit of Quantification (Ratio + 10 SD)/a; CVr: Coefficients of variation repeatability; CVR: coefficients of variation Reproducibility; Conc: Concentration level used for repeatability and reproducibility measurements.

**Supplementary Table 2:** Distribution of participants by KDIGO G-stage at baseline, SKIPOGH study, Lausanne, Switzerland.

| **KDIGO G-stage** | **eGFR(mL/min/1.73m²)** | **n (%)** |
| --- | --- | --- |
| G1 | ≥90 | 669 (67.71%) |
| G2 | 60–89 | 287 (29.05%) |
| G3a | 45–59 | 25 (2.53%) |
| G3b | 30–44 | 7 (0.71%) |
| G4 | 15–29 | 0 (0.00%) |
| G5 | <15 | 0 (0.00%) |

KDIGO G-stage: Kidney Disease: Improving Global Outcomes glomerular filtration rate stages.

**Supplementary Table 3:** Collinearity diagnostics (variance inflation factor and tolerance) for 24 log-transformed plasma metals and trace elements, SKIPOGH study, Lausanne, Switzerland.

| **Metals/trace elements** | **VIF** | **Tolerance** |
| --- | --- | --- |
| Li | 1.17 | 0.857 |
| Be | 1.11 | 0.901 |
| Al | 1.18 | 0.851 |
| V | 1.39 | 0.719 |
| Cr | 1.67 | 0.600 |
| Mn | 1.30 | 0.768 |
| Co | 1.09 | 0.919 |
| Ni | 1.66 | 0.604 |
| Cu | 1.34 | 0.748 |
| Zn | 1.44 | 0.696 |
| As | 1.28 | 0.781 |
| Se | 1.47 | 0.682 |
| Mo | 1.11 | 0.904 |
| Pd | 1.43 | 0.700 |
| Ag | 1.30 | 0.770 |
| Cd | 1.46 | 0.684 |
| Sn | 1.61 | 0.623 |
| Sb | 1.63 | 0.614 |
| I | 1.77 | 0.564 |
| Pt | 1.44 | 0.695 |
| Hg | 1.31 | 0.763 |
| Tl | 1.09 | 0.919 |
| Pb | 1.30 | 0.771 |
| Bi | 1.47 | 0.682 |

Variance inflation factors (VIFs) and Tolerance values were calculated by fitting all 24 log-transformed plasma metal/trace element concentrations into a single linear regression model with a randomly generated outcome (Gaussian distribution), as collinearity diagnostics are independent of outcome variables.

**Supplementary Table 4:** Characteristics of participants included vs. excluded from the analysis. SKIPOGH study, Lausanne, Switzerland.

| **Variables** | **Included (n=988)** | **Excluded (n=141)** | ***P-value*** |
| --- | --- | --- | --- |
| Age, years | 47.0 ± 17.6 | 50.7 ± 16.3 | **0.017** |
| Female sex, % | 517 (52.3) | 72 (51.1) | 0.779 |
| Education level, % |  |  | 0.329 |
| High | 360 (37.0) | 42 (31.6) |  |
| Middle | 437 (44.9) | 61 (45.9) |  |
| Low | 176 (18.1) | 30 (22.5) |  |
| Marital status, % |  |  | 0.551 |
| Living alone | 315 (32.0) | 40 (29.4) |  |
| Living in couple | 671 (68.0) | 96 (70.6) |  |
| Smoking status, % |  |  | 0.323 |
| Never | 442 (45.0) | 56 (41.8) |  |
| Former | 297 (30.2) | 49 (36.6) |  |
| Current | 243 (24.8) | 29 (21.6) |  |
| Alcohol consumption (%) |  |  | **0.005** |
| None | 364 (36.9) | 43 (30.5) |  |
| 1-13/week | 435 (44.0) | 60 (42.6) |  |
| 14-27/week | 106 (10.7) | 13 (9.2) |  |
| 28+/week | 83 (8.4) | 25 (17.7) |  |
| BMI group, % |  |  | 0.280 |
| Normal | 856 (86.6) | 125 (89.9) |  |
| Obese | 132 (13.4) | 14 (10.1) |  |
| Hypertension, % | 303 (30.8) | 43 (32.1) | 0.755 |
| Diabetes, % | 42 (4.3) | 9 (7.0) | 0.163 |
| Blood creatine, umol/L | 73.5 ± 14.2 | 74.5 ± 14.1 | 0.457 |
| C-reactive protein (mg/L) | 1.5[1.0-2.6] | 1.5[1.0-3.0] | 0.553 |
| Vitamin D (nmol/L) | 93.0 ± 33.8 | 92.0 ± 32.1 | 0.745 |
| Urinary albumin (mg/L) | 4.0[1.5-8.0] | 5.0[2.0-10.0] | 0.066 |
| UACR categories |  |  | 0.649 |
| A1 | 921 (95.2) | 132 (96.4) |  |
| A2 | 42 (4.4) | 4 (2.9) |  |
| A3 | 4 (0.4) | 1 (0.7) |  |
| Kidney function (%) |  |  | 0.510 |
| Normal | 916 (92.7) | 131 (94.2) |  |
| CKD | 72 (7.3) | 8 (5.8) |  |
| eGFR, mL/min/1.73m^2^ | 96.8 ± 17.9 | 93.2 ± 18.0 | **0.030** |

Results are expressed as the number of participants (column percentage) for categorical variables and as average ± standard deviation or median and [interquartile range] for continuous variables. Between-group comparisons were performed using chi-square for categorical variables and Student’s t-test or Kruskal-Wallis test for continuous variables. Abbreviations: CKD, chronic kidney disease; BMI, body mass index; eGFR, estimated glomerular filtration rate.

**Supplementary Table 5:** Sensitivity analysis (restricted to participants with eGFR ≥ 60 mL/min/1.73m²): Associations of plasma element concentrations with continuous eGFR and log-transformed albuminuria (log-UACR), SKIPOGH study, Lausanne, Switzerland.

| **Metals/trace elements** | **eGFR**  **Coefficient (95% CI)** | ***P* value** | **log-UACR**  **Coefficient (95% CI)** | ***P* value** |
| --- | --- | --- | --- | --- |
| Li | 0.11 (-0.59, 0.82) | 0.752 | 0.00 (-0.07, 0.06) | 0.903 |
| Be | 0.50 (-0.10, 1.11) | 0.102 | -0.03 (-0.09, 0.04) | 0.372 |
| Al | 0.35 (-0.33, 1.03) | 0.309 | -0.08 (-0.15, 0.00) | **0.039** |
| V | 0.84 (-3.29, 4.97) | 0.690 | -0.05 (-0.34, 0.24) | 0.728 |
| Cr | 0.31 (-0.53, 1.16) | 0.463 | -0.04 (-0.12, 0.04) | 0.275 |
| Mn | 0.83 (-2.06, 3.72) | 0.573 | 0.01 (-0.24, 0.26) | 0.926 |
| Co | 0.48 (-0.67, 1.63) | 0.411 | 0.05 (-0.04, 0.15) | 0.282 |
| Ni | -0.02 (-1.22, 1.17) | 0.967 | 0.05 (-0.05, 0.16) | 0.340 |
| Cu | -2.66 (-5.95, 0.63) | 0.113 | 0.27 (-0.03, 0.56) | 0.081 |
| Zn | -5.65 (-10.48, -0.82) | **0.022** | -0.20 (-0.67, 0.28) | 0.409 |
| As | -0.22 (-0.89, 0.44) | 0.505 | 0.03 (-0.02, 0.09) | 0.252 |
| Se | -5.02 (-10.51, 0.46) | 0.072 | 0.11 (-0.29, 0.50) | 0.596 |
| Mo | -0.47 (-2.33, 1.39) | 0.619 | 0.22 (0.07, 0.38) | **0.005** |
| Pd | -0.41 (-1.56, 0.75) | 0.488 | 0.00 (-0.12, 0.12) | 0.987 |
| Ag | 0.51 (-0.19, 1.22) | 0.154 | -0.01 (-0.07, 0.04) | 0.680 |
| Cd | -1.22 (-3.62, 1.17) | 0.315 | 0.11 (-0.07, 0.28) | 0.229 |
| Sn | -0.84 (-1.46, -0.22) | **0.008** | -0.02 (-0.08, 0.04) | 0.456 |
| Sb | -0.32 (-0.90, 0.25) | 0.272 | 0.01 (-0.03, 0.05) | 0.543 |
| I | -0.57 (-1.69, 0.55) | 0.314 | 0.12 (0.04, 0.20) | **0.004** |
| Pt | -0.35 (-1.45, 0.75) | 0.529 | 0.02 (-0.05, 0.08) | 0.650 |
| Hg | -0.15 (-1.40, 1.11) | 0.819 | -0.06 (-0.16, 0.03) | 0.194 |
| Tl | -1.93 (-3.77, -0.10) | 0.039 | -0.08 (-0.21, 0.05) | 0.228 |
| Pb | -1.06 (-2.24, 0.12) | 0.079 | -0.11 (-0.20, -0.02) | **0.017** |
| Bi | -0.27 (-0.93, 0.38) | 0.416 | 0.05 (-0.01, 0.10) | 0.111 |

Results are presented as linear regression coefficients with 95% confidence intervals (CI), based on multivariable linear regression models. All models were adjusted for age, sex, education level, marital status, smoking status, alcohol consumption, physical activity, obesity, hypertension, diabetes, vitamin D, C-reactive protein, study center, and accounted for familial clustering using robust standard errors. eGFR was analyzed as a continuous variable, and albuminuria was log-transformed (log-UACR) before analysis. Elements were modeled continuously per one-unit increase in log-transformed plasma concentrations.

**Supplementary Table 6:** Sensitivity analysis using Firth logistic regression: associations between single plasma element concentrations and CKD, SKIPOGH study, Lausanne, Switzerland.

| **Metals/trace elements** | **OR (95% CI)** | ***P* value** |
| --- | --- | --- |
| Li | 0.98 (0.78, 1.23) | 0.859 |
| Be | 0.95 (0.77, 1.17) | 0.635 |
| Al | 0.78 (0.61, 0.99) | **0.044** |
| V | 0.71 (0.15, 3.46) | 0.671 |
| Cr | 0.94 (0.70, 1.26) | 0.664 |
| Mn | 2.71 (0.86, 8.52) | 0.088 |
| Co | 1.16 (0.78, 1.73) | 0.466 |
| Ni | 1.19 (0.81, 1.75) | 0.378 |
| Cu | 4.25 (1.07, 16.88) | **0.040** |
| Zn | 1.04 (0.14, 7.70) | 0.967 |
| As | 1.20 (0.97, 1.48) | 0.086 |
| Se | 0.31 (0.05, 1.79) | 0.191 |
| Mo | 2.49 (1.26, 4.94) | **0.009** |
| Pd | 1.36 (0.85, 2.17) | 0.195 |
| Ag | 0.76 (0.57, 1.00) | 0.054 |
| Cd | 1.26 (0.53, 2.99) | 0.598 |
| Sn | 0.92 (0.68, 1.25) | 0.587 |
| Sb | 1.25 (0.91, 1.72) | 0.170 |
| I | 1.88 (1.19, 2.97) | **0.007** |
| Pt | 1.18 (0.69, 2.02) | 0.536 |
| Hg | 0.75 (0.48, 1.20) | 0.230 |
| Tl | 0.95 (0.45, 2.04) | 0.901 |
| Pb | 1.03 (0.67, 1.59) | 0.882 |
| Bi | 1.05 (0.79, 1.39) | 0.742 |

Firth logistic regression was used to reduce small-sample bias due to the limited number of CKD cases. Models were adjusted for age, sex, education, marital status, smoking, alcohol intake, obesity, hypertension, diabetes, vitamin D, C-reactive protein, physical activity, and study center. Plasma concentrations were log-transformed. ORs are reported per 1-unit increase in log concentration.

**Supplementary Table 7:** Sensitivity analysis: Associations of plasma element concentrations with CKD defined by eGFR <60 mL/min/1.73m² only (excluding albuminuria), SKIPOGH study, Lausanne, Switzerland.

| **Metals/trace elements** | **OR (95% CI)** | ***P* value** |
| --- | --- | --- |
| Li | 1.08 (0.83, 1.40) | 0.588 |
| Be | 1.00 (0.67, 1.51) | 0.994 |
| Al | 0.83 (0.53, 1.31) | 0.433 |
| V | 0.10 (0.01, 1.84) | 0.122 |
| Cr | 0.58 (0.42, 0.82) | **0.002** |
| Mn | 1.33 (0.21, 8.45) | 0.762 |
| Co | 0.76 (0.38, 1.51) | 0.434 |
| Ni | 2.17 (1.01, 4.67) | **0.048** |
| Cu | 0.21 (0.01, 3.54) | 0.277 |
| Zn | 0.14 (0.00, 9.87) | 0.362 |
| As | 1.20 (0.90, 1.61) | 0.207 |
| Se | 0.16 (0.01, 1.93) | 0.151 |
| Mo | 3.99 (1.18, 13.51) | **0.026** |
| Pd | 0.99 (0.51, 1.94) | 0.981 |
| Ag | 1.14 (0.69, 1.88) | 0.602 |
| Cd | 1.76 (0.48, 6.42) | 0.395 |
| Sn | 0.84 (0.44, 1.63) | 0.612 |
| Sb | 1.79 (1.27, 2.52) | **0.001** |
| I | 1.97 (1.18, 3.28) | **0.010** |
| Pt | 1.31 (0.59, 2.90) | 0.513 |
| Hg | 0.93 (0.45, 1.94) | 0.846 |
| Tl | 0.45 (0.13, 1.56) | 0.209 |
| Pb | 1.45 (0.73, 2.88) | 0.292 |
| Bi | 0.94 (0.65, 1.37) | 0.754 |

To test robustness to outcome definition, CKD was redefined based on eGFR <60 only. Logistic regression models were adjusted for the same covariates as in the main analysis. Plasma element concentrations were log-transformed. ORs are reported per 1-unit increase in log concentration, with robust standard errors clustered by family.

**Supplementary Table 8:** Sensitivity analysis excluding top 1% values of plasma aluminum (Al) and silver (Ag): associations with eGFR and CKD, SKIPOGH study, Lausanne, Switzerland.

| **Elements** | **Main Estimate**  **Coefficient / OR**  **(95% CI)** | ***P* value** | **After Excluding Top 1% Coefficient / OR**  **(95% CI)** | ***P* value** |
| --- | --- | --- | --- | --- |
| **eGFR** |  |  |  |  |
| Al | 0.32 (–0.44, 1.09) | 0.403 | 0.24 (–0.58, 1.06) | 0.565 |
| Ag | 0.51 (–0.26, 1.28) | 0.195 | 0.53 (–0.24, 1.30) | 0.174 |
| **CKD** |  |  |  |  |
| Al | 0.77 (0.60, 0.99) | **0.041** | 0.78 (0.60, 1.01) | 0.061 |
| Ag | 0.75 (0.58, 0.97) | **0.031** | 0.69 (0.54, 0.88) | **0.003** |

Results show the main effect estimates and those after excluding participants with plasma Al or Ag concentrations above the 99th percentile. eGFR was modeled using linear regression (coefficients), and CKD using logistic regression (odds ratios). Models were adjusted for age, sex, education level, marital status, smoking, alcohol consumption, physical activity, obesity, hypertension, diabetes, vitamin D, CRP, and study center, with robust standard errors clustered by family code.

**Supplementary Table 9:** Sensitivity analysis excluding plasma elements with >10% of measurements below the limit of detection (LOD): associations with eGFR and CKD, SKIPOGH study, Lausanne, Switzerland.

| **Metals/trace elements** | **% < LOD** | **Main Estimate**  **Coefficient / OR**  **(95% CI)** | ***P* value** | **After Excluding LOD>10% Coefficient / OR**  **(95% CI)** | ***P* value** |
| --- | --- | --- | --- | --- | --- |
| **eGFR** |  |  |  |  |  |
| Li | 15.89% | 0.00 (-0.72, 0.73) | 0.993 | -0.203 (-1.18, 0.78) | 0.684 |
| Be | 35.53% | 0.44 (-0.21, 1.08) | 0.187 | -0.033 (-1.35, 1.29) | 0.961 |
| Al | 64.27% | 0.32 (-0.44, 1.09) | 0.403 | 3.18 (0.39, 5.96) | 0.025 |
| Cr | 39.57% | 0.63 (-0.29, 1.55) | 0.181 | 1.57 (-0.59, 3.72) | 0.153 |
| Ni | 11.94% | -0.54 (-1.83, 0.76) | 0.416 | -0.48 (-2.93, 1.96) | 0.697 |
| Ag | 26.11% | 0.51 (-0.26, 1.28) | 0.195 | 0.66 (-0.67, 1.98) | 0.332 |
| Sn | 14.17% | -0.76 (-1.46, -0.06) | **0.034** | -0.54 (-1.77, 0.69) | 0.387 |
| Pt | 25.71% | -0.34 (-1.49, 0.82) | 0.566 | 1.04 (-0.81, 2.89) | 0.269 |
| Bi | 23.38% | -0.25 (-0.98, 0.49) | 0.509 | 0.37 (-1.01, 1.76) | 0.594 |
| **CKD** |  |  |  |  |  |
| Li | 15.89% | 0.98 (0.78, 1.22) | 0.823 | 1.00 (0.77, 1.29) | 0.986 |
| Be | 35.53% | 0.95 (0.77, 1.19) | 0.667 | 1.40 (0.88, 2.22) | 0.158 |
| Al | 64.27% | 0.77 (0.60, 0.99) | **0.041** | 0.54 (0.16, 1.78) | 0.309 |
| Cr | 39.57% | 0.94 (0.72, 1.22) | 0.632 | 1.03 (0.46, 2.27) | 0.946 |
| Ni | 11.94% | 1.22 (0.85, 1.76) | 0.274 | 1.19 (0.58, 2.44) | 0.626 |
| Ag | 26.11% | 0.75 (0.58, 0.97) | **0.031** | 0.73 (0.41, 1.30) | 0.281 |
| Sn | 14.17% | 0.92 (0.70, 1.21) | 0.548 | 0.91 (0.57, 1.47) | 0.708 |
| Pt | 25.71% | 1.20 (0.85, 1.69) | 0.297 | 0.83 (0.39, 1.75) | 0.619 |
| Bi | 23.38% | 1.06 (0.86, 1.31) | 0.593 | 0.94 (0.59, 1.52) | 0.811 |

Estimates are presented as regression coefficients (eGFR, linear model) or odds ratios (CKD, logistic model) with 95% confidence intervals. Values below the limit of detection (LOD) were excluded for sensitivity analysis when >10% of the measurements were censored. All models were adjusted for covariates listed in the main analysis.

**Supplementary Figure 1:** Selection of participants from SKIPOGH 2009–2013. eGFR: estimated glomerular filtration rate. SKIPOGH study, Lausanne, Switzerland.

**Supplementary Figure 2:** Dose-response relationships between 24 plasma elements and eGFR.

**Supplementary Figure 3:** Pearson correlation matrix of log-transformed 24 plasma elements.

**
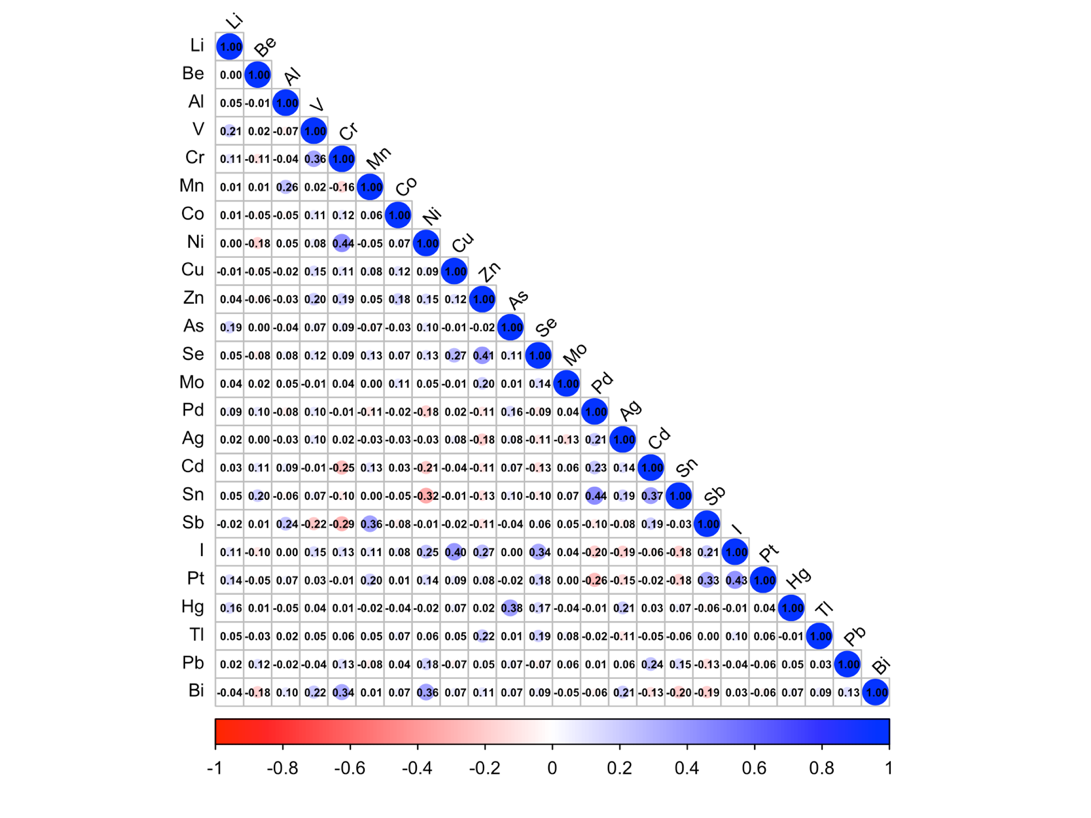
**

Pearson correlation matrix between 24 log-transformed plasma metal and trace element concentrations. The size and color of the circles indicate the strength and direction of the pairwise correlation coefficients (r). Blue represents positive correlations, and red represents negative correlations.

**Supplementary Figure 4:** Dose-response relationships between 24 plasma elements and CKD.
